# A Data-Driven Approach to Estimating Occupational Inhalation Exposure Using Workplace Compliance Data

**DOI:** 10.1021/acs.est.2c08234

**Published:** 2023-03-30

**Authors:** Jeffrey M. Minucci, S. Thomas Purucker, Kristin K. Isaacs, John F. Wambaugh, Katherine A. Phillips

**Affiliations:** † Center for Public Health and Environmental Assessment, Office of Research and Development, US Environmental Protection Agency, 109 TW Alexander Drive, Durham, North Carolina 27709, United States; ‡ Center for Computational Toxicology and Exposure, Office of Research and Development, US Environmental Protection Agency, 109 TW Alexander Drive, Durham, North Carolina 27709, United States

**Keywords:** occupational exposure, high-throughput, screening, hierarchical model, Bayesian, air monitoring

## Abstract

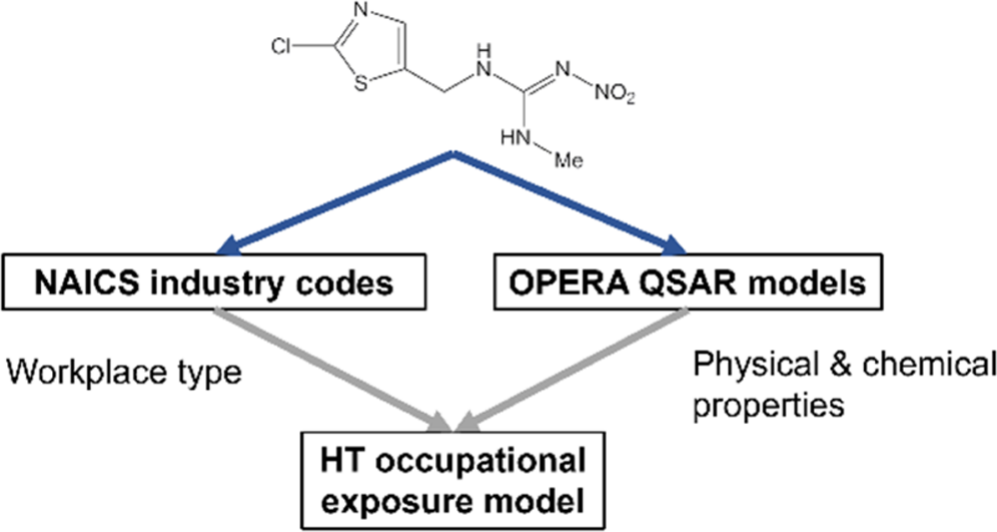

A growing list of chemicals are approved for production and use in the United States and elsewhere, and new approaches are needed to rapidly assess the potential exposure and health hazard posed by these substances. Here, we present a high-throughput, data-driven approach that will aid in estimating occupational exposure using a database of over 1.5 million observations of chemical concentrations in U.S. workplace air samples. We fit a Bayesian hierarchical model that uses industry type and the physicochemical properties of a substance to predict the distribution of workplace air concentrations. This model substantially outperforms a null model when predicting whether a substance will be detected in an air sample, and if so at what concentration, with 75.9% classification accuracy and a root-mean-square error (RMSE) of 1.00 log_10_ mg m^–3^ when applied to a held-out test set of substances. This modeling framework can be used to predict air concentration distributions for new substances, which we demonstrate by making predictions for 5587 new substance-by-workplace-type pairs reported in the US EPA’s Toxic Substances Control Act (TSCA) Chemical Data Reporting (CDR) industrial use database. It also allows for improved consideration of occupational exposure within the context of high-throughput, risk-based chemical prioritization efforts.

## Abstract 2

High-throughput occupational exposure models are needed to screen tens of thousands of chemicals for possible risk. This study demonstrates a Bayesian hierarchical modeling approach to predict workplace chemical concentrations in air based on physicochemical properties and industry classification.

## Introduction

The vast number of chemicals approved for use in commerce has necessitated the development of high-throughput computational techniques that can identify potentially high-risk chemicals for further scrutiny.^1−3^ These techniques typically estimate chemical exposure for the general population by mining data sources such as consumer product formulations^4−6^ and biomonitoring data,^7−9^ screening for suspect compounds,^10,11^ and applying various machine learning and/or probabilistic meta-models that balance contributions from a variety of exposure pathways.^12−14^ However, in the United States, the 2016 Frank Lautenberg Chemical Safety for the 21st Century Act specifically mandates the protection of highly exposed subpopulations, whose exposure risk may not be adequately characterized by the broad, cross-sectional datasets typically available as inputs for high-throughput models. As a result, screening-level models are needed to estimate exposure for tens of thousands of chemicals across many specific subpopulations, but data availability remains a key challenge when developing such models.^3^

Workers are a specific subpopulation at risk for far greater exposure than the general population to a wide array of chemicals. As a result, occupational exposure is estimated to cause over 290 000 deaths globally each year.^15^ Because working-age adults spend a large proportion of their time at places of work, characterizing chemical exposure in these environments is key to understanding the full picture of chemical exposures received by these individuals. However, the types and amounts of chemicals present in work environments vary dramatically across and within industry types. Even within a single workplace, chemical exposure may depend on a worker’s specific tasks, job assignment, and schedule.^16,17^ In addition to the challenge of high variability between and within workplaces, data on the production volume of each substance and how they are utilized in industrial processes may be unavailable or highly censored if this information is considered confidential or protected business information.^18^

High-throughput occupational exposure must be handled very differently than general population exposure, or even typical subpopulation exposures in that every occupation must be evaluated differently than other occupations. For example, modeling exposure for a specific age or ethnic group subpopulation requires changing the value of parameters representing the general behavior or bio-relevant information for that group. But the uniqueness of every job (e.g., its environment and chemical exposure scenarios) does not allow for easy substitution of group-specific information. Luckily, monitoring data are available to address occupation-to-occupation differences. Workplace monitoring data coming from sources such as dermal wipes or air sampling in work areas or the breathing zone of individual workers can be used to set occupational exposure limits and assess compliance with those limits. Occupational exposure limits are set to mitigate risk and are defined specific to a single chemical for a single exposure route (e.g., dermal, ingestion, inhalation, ocular). These limits allow employers to take preventative measures to limit exposure through use of personal protective equipment (PPE) or adjustments made to worker activities.^19,20^ While such monitoring data has been used previously to inform occupational exposure estimates for select occupation–chemical couples,^21^ it has not been generalized to the myriad of occupation and chemical combinations.

When monitoring data are available, they can be used to predict exposures using modeling tools such as the Chemical Screening Tool for Exposures and Environmental Releases (ChemSTEER). ChemSTEER is a screening-level tool developed by the US EPA’s Office of Pollution Prevention and Toxics that provides a means to estimate worker exposure via several inhalation and dermal exposure models.^22,23^ These models require as inputs some information on the amount of the substance present in the worker’s environment, e.g., as an air concentration or a vapor generation rate,^24,25^ which can be derived from monitoring data. When such monitoring data is not available, ChemSTEER includes a number of “release models” that can be used to estimate chemical releases that occur during specific activities, for example, loading or unloading a liquid from a container or cleaning a container with solid residues.^26^ However, a key challenge to developing high-throughput occupational exposure modeling that can screen tens of thousands of substances is that we typically lack data on both the amount of each substance that might be present in a workplace environment and the specific activities that might be carried out using the substance.^3,27^

Here, we present a data-driven statistical approach to estimating potential workplace air concentrations based on workplace type and the physicochemical properties of a substance. We focus our analysis on organic substances, where quantitative structure–activity/property relationship (QSAR/QSPR) models can be applied to predict physicochemical properties. We leverage monitoring data from the publicly available Occupational Safety and Health Administration (OSHA) Chemical Exposure Health Data, a dataset containing industrial hygiene samples taken by OSHA compliance officers in workplaces between 1984 and 2018.^28^ We focused our analysis on air samples because they represent the bulk of the data available, and, for most chemicals, inhalation is the primary exposure route at workplaces.^16^ Using these data, we trained a screening model using a Bayesian hierarchical modeling approach that accounts for similarities between workplace types, along with physicochemical properties predicted from chemical structure.

## Methods

### Workplace Air Concentration Data

We used the publicly available OSHA Chemical Exposure Health Data as a source for air concentrations of chemicals in United States workplaces.^28^ The dataset consists of samples taken at workplaces by OSHA compliance officers between 1984 and 2018. For this analysis, we included only air samples of two types: “personal” samples (*N* = 1 524 921) taken in the immediate breathing zone of a worker, and “area” samples (*N* = 61 028) taken in zones representative of an industrial process or multiple workers’ exposure. Blanks and samples of other types (e.g., dermal wipes, bulk substance samples) were removed. All sample results were converted to units of log_10_ mg m^–3^, using molecular weight when required, and all types of sampling methods (e.g., instantaneous samples, time-integrated samples) were treated identically. We dropped any samples that could not be converted to a concentration, for example, samples with the units “fibers/cc”, or those that were reported in mass units without providing the amount of air sampled (85 821 samples dropped). The majority of such cases were for inorganic substances (e.g., asbestos, silica, lead) which were outside the scope of this model. Workplace type was reported in the OSHA data using two different hierarchical classification systems, the North American Industry Classification System (NAICS) for years 2002–2018^29^ and the Standard Industrial Classification (SIC) for years 1984–2002.^30^ We mapped the classification system used in each year’s data to that of the 2017 NAICS code system (the most recent at the time of the analysis) using NAICS concordance tables^31^ and identified the three-digit “subsectors” (*j* = 75) and two-digit “sectors” (*k* = 19) for each workplace. If a workplace could not be matched to a single 2017 NAICS sector and subsector, the associated data were removed from the analysis (259 299 samples dropped).

### Structure Matching and Physicochemical Properties

To determine the molecular structure of each substance recorded in the air sampling data, we first matched the substance names recorded by OSHA to specific chemical structures and preferred names in the US EPA Distributed Structure-Searchable Toxicity (DSSTox) Database^32^ using the synonym search feature on the US EPA CompTox Chemicals Dashboard.^33,34^ A specific chemical structure was defined by a DSSTox substance identifier (DTXSID). In cases where no synonymous structure was found, we manually curated structure matches using the PubChem search function^35^ and dropped any samples with substance names that could not be matched to a single structure (DTXSID), such as mixtures of indeterminate composition (64/648 substances).

We then used the OPEn structure–activity/property Relationship App (OPERA) v2.5 suite of QSAR models^36^ to predict physicochemical properties for each organic substance in the OSHA dataset. Although many well-known chemicals have empirically measured physicochemical property information available, we chose to use only OPERA-predicted properties to streamline the high-throughput workflow of the model and improve applicability for large chemical lists with many novel or poorly described chemicals. For sets of highly correlated properties (|Pearson’s *r*| > 0.85), we kept one property in the analysis based on expert judgment and dropped all others, resulting in six predictors out of an initial 12 (Table 1). Properties were then scaled and centered to have means of zero and unit variance. Physicochemical properties were not predicted for inorganic substances because they are outside the domain of the QSAR models.

**Table 1:** List of OPERA-Predicted Physicochemical Properties and Their Distributions across the Substances in the OSHA Workplace Air Dataset prior to Centering and Scaling

physicochemical property	abbreviation	unit	mean	range
log octanol–water partition coefficient	log p	unitless	1.98	–3.61 to 9.23
boiling point	bp	°C	212.3	–149.0 to 532.5
log Henry’s law constant	log hl	log atm-m^3^ mol^–1^	–5.48	–11.25 to 1.06
HPLC retention time	rt	min	8.34	0.00–52.9
log OH rate constant^a^	log oh	log cm^3^ molecule-s^–1^	–11.2	–15.9 to −9.63
log soil adsorption coefficient	log koc	log L kg^–1^	2.19	0.22–6.41

a OH rate constant represents the rate constant for the atmospheric reaction between photochemically produced hydroxyl radicals and the compound of interest.

### Training and Test Sets

**Figure 1:**
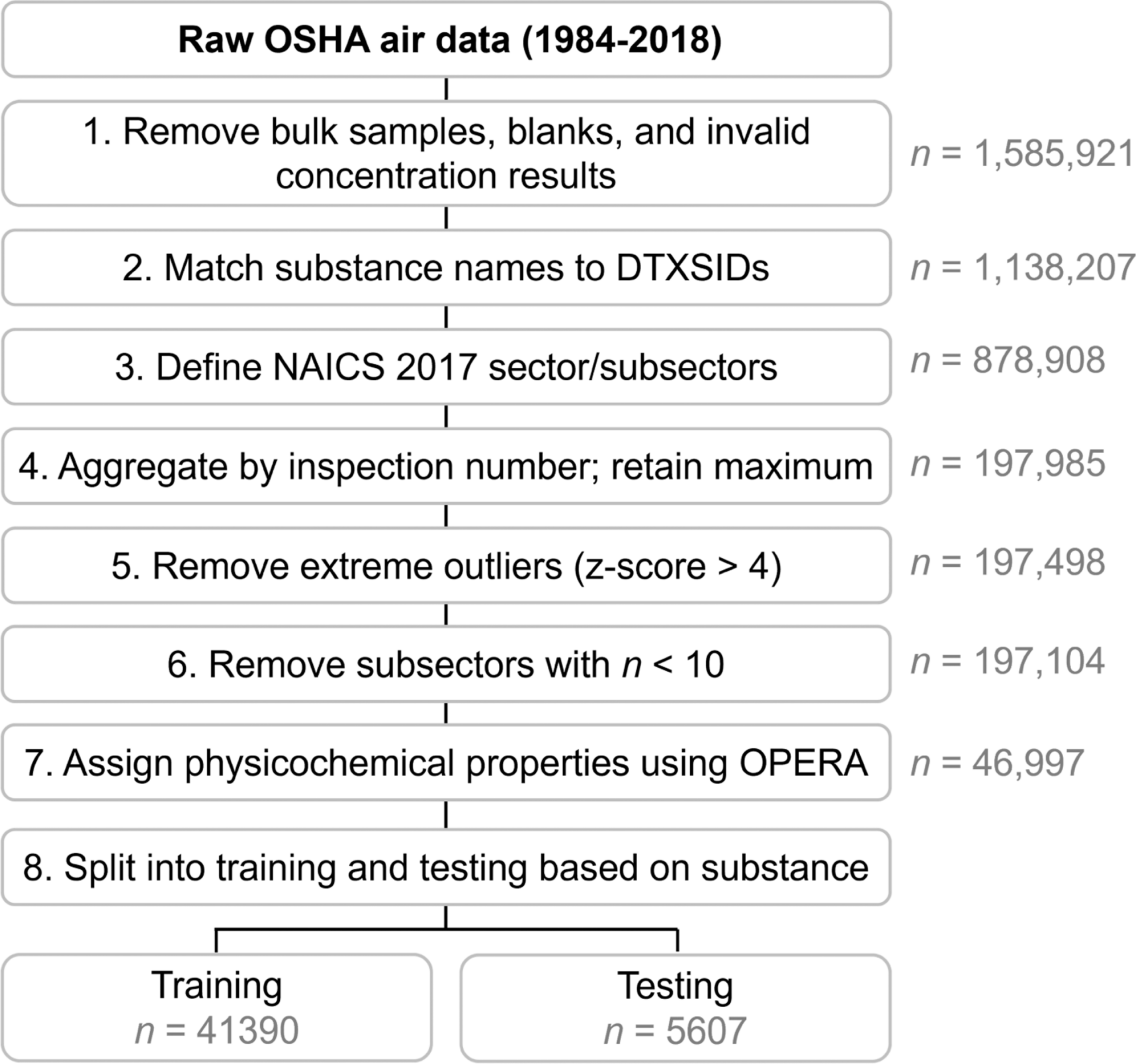
Diagram of data processing workflow for Occupational Safety and Health Administration (OSHA) Chemical Exposure Health Safety Data. Numbers in gray denote the sample size after the step is completed.

We split the dataset into a training set for model fitting and a test set to assess the performance of our model on new data. However, some additional pre-processing steps were carried out before the training/test split. First, because multiple samples were sometimes taken within a single sampling effort, we aggregated air concentrations by inspection number, taking the maximum observed value to characterize the worst-case scenario of worker exposure which resulted in 197 985 observations. The median number of samples per inspection was 3, with a maximum and minimum of 281 and 1, respectively. Next, we excluded extreme outliers that had a *z*-score greater than 4 compared with other observations of the same substance (487 observations dropped). Then, we dropped all data for NAICS workplace subsectors that had less than 10 detects (19 subsectors dropped, 394 observations dropped) and for substances without physicochemical property predictions (i.e., inorganic compounds; 150 107 observations dropped). We then randomly selected 10% of the substances present in the OSHA workplace air monitoring dataset (58 substances, 5607 samples) and held out all data associated with these substances to form the test set, while the remaining 90% of substances made up the training set (527 substances, 41 390 samples). By splitting the dataset by substance, we ensured that the test set contained a combination of physicochemical properties that our model had never seen during the fitting process. The final datasets used for model training and testing are provided in Tables S1 and S2. A diagram of the data processing workflow is provided in Figure 1.

### Bayesian Hierarchical Model Structure

Because the workplace air concentration data had a high proportion of nondetects reported (39%), we used a two-stage (or hurdle) model where the first model stage predicts whether a substance will be detected or not, and the second stage predicts the air concentration for detected substances.^37^ For the first stage, we converted the workplace air data to a binary detect/nondetect response variable and fit a Bayesian hierarchical logistic regression model. For the second stage, we fit a similarly structured, but nonlogistic, Bayesian hierarchical regression model to a continuous air concentration response variable, using only data where a substance was detected.

In both models, we included the type of workplace where sampling occurred as a hierarchical intercept term based on NAICS sector and subsector. The model equations (eq 1) include an intercept, *β*_1*j*_, for each subsector *j*, which was sampled from a Student’s *T* distribution where the mean varies based on sector *k*. This hierarchical structure allowed us to consider differences in exposure between subsectors, while accounting for the fact that subsectors in the same sector may have similar exposure patterns. It also allowed subsectors with few observations to be influenced by data from other subsectors in the same sector.

Both models also incorporated OPERA-predicted physicochemical properties as linear regression terms with coefficients *β*_2*m*_ and predictors *X*_*m*_. They included regression terms for the main effects of each physicochemical property plus all possible two-way interactions. The model structure for both models was
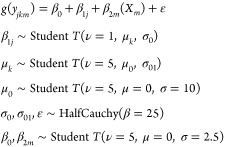
1where *g*(*y*) is the logit link function for the first-stage detect/nondetect model and the identity function for the second-stage concentration model. All priors and hyper-priors were chosen to be weakly informative for generalized linear regression.^38^ Student’s *T* prior parameters were ν, *μ*, and *σ*, which represent the degrees of freedom, location, and scale of the distribution, respectively. Model random error (ε) and Student’s *T* scale parameters σ_0_ and σ_01_ were modeled with a half-Cauchy prior with β representing the scale parameter. Model fitting was performed in Python using full-rank automatic differentiation variational inference (ADVI) as implemented in the pyMC3 package.^39^ Model code and scripts to run the analysis are available at https://github.com/USEPA/ht_occupational or https://doi.org/10.5281/zenodo.7737239. The total run time for model fitting is about 30 min on a 16-thread processor. The fitted model predicts the probability of detection, and if detected, predicts an air concentration, for a chemical with a given set of physicochemical properties, in a workplace with a given NAICS sector/subsector that must be one of the NAICS codes that occurred in the OSHA monitoring dataset. That is, the model makes predictions for a specified substance-by-workplace pair.

### Applying the Fit Model to Screen Novel Substance and Workplace Combinations

To further demonstrate how our model fit to the OSHA monitoring data could be used to screen new substance and workplace combinations where monitoring data is lacking, we leveraged data from the US EPA’s Toxic Substances Control Act (TSCA) Chemical Data Reporting (CDR) database, 2016 cycle.^40^ The CDR rule implemented under TSCA requires manufacturers to provide information such as the types of chemicals, amounts produced or imported, the uses of that chemical, as well as the industrial sectors to the US EPA to provide exposure-relevant data to federal risk assessors (Code of Federal Regulations, Title 40, Chapter 1, Subchapter R, Part 711). From the 2016 CDR data, we collected substance-by-NAICS sector/subsector combinations that were not included in the OSHA data analysis and used our model to predict a detection probability and an air concentration for detects. For more details on CDR data processing, see Section S1.

## Results

### Model Performance on Training and Test Sets

The first stage of our model was able to predict whether a given substance would be detected in a workplace air sampling effort with 69.6% classification accuracy on the training set. The area under the receiver operating characteristic curve (AUC), which considers the imbalance between detect and nondetect frequencies, was 0.72. In comparison, a null model which considered only the overall detection rate had 47.5% classification accuracy and 0.50 AUC on the training set. The second stage of our model, trained on samples where the substance was detected in workplace air, had a root-mean-square error (RMSE) of 0.94 log_10_ mg m^–3^ on the training data, while the null model had an RMSE of 1.48 log_10_ mg m^–3^.

**Figure 2:**
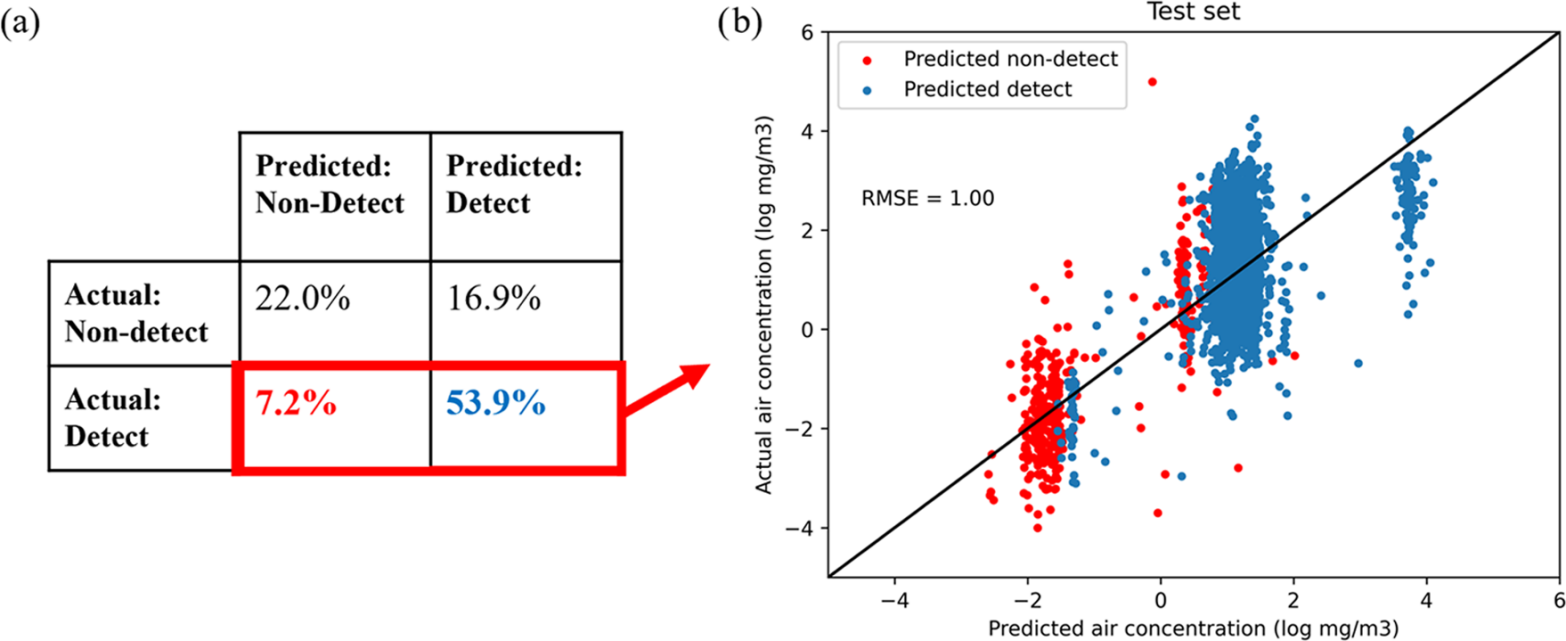
(a) Confusion matrix of two-step model performance on the test set (*N* = 5641) and (b) actual vs predicted workplace air concentration (log_10_ mg m^–3^) for empirically detected air samples.

When applying our combined hurdle model to the novel substances in the test set, we were able to predict whether a substance would be detected with 75.9% accuracy. Our model was more accurate when predicting detects than nondetects, which was reflected in an 88.2% true positive rate  and a 56.6% true negative rate  (Figure 2a). AUC on the test set was 0.79, similar to the value for the training set. In comparison, predictions from the null model had a 48.1% classification accuracy and an AUC of 0.50. For samples that were empirically detected in workplaces and also predicted to be detected (true positives), the air concentration part of our model had an RMSE of 1.00 log_10_ mg m^–3^, while the null model had an RMSE of 1.43 log_10_ mg m^–3^ (Figure 2b). For true positives, the model prediction was within 1 order of magnitude of the empirically measured air concentration in mg m^–3^ for 65.1% of cases. In comparison, the total range of the empirical data was about 11 orders of magnitude.

### Relationship between Physicochemical Properties and Workplace Air Monitoring

**Figure 3:**
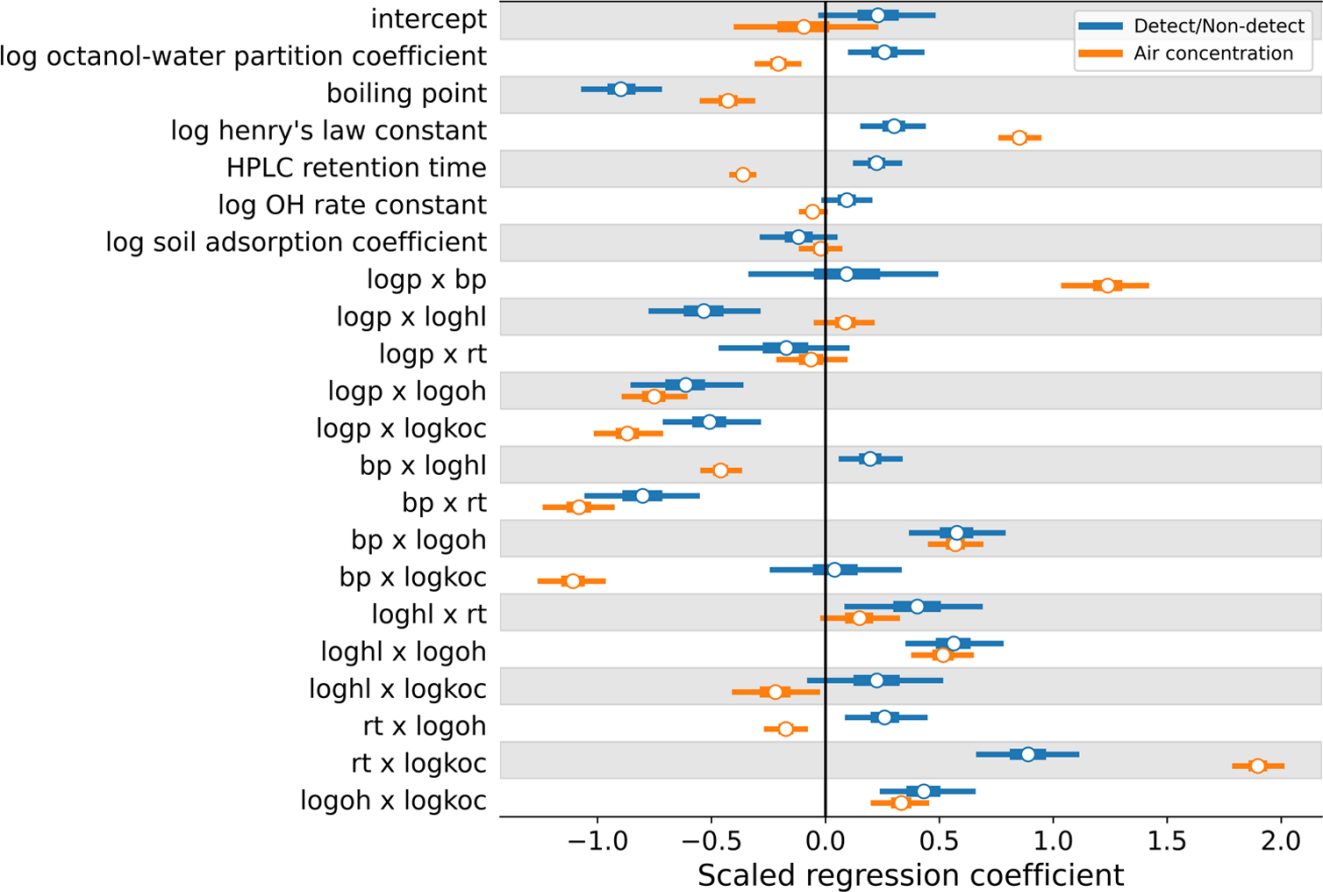
Distribution of coefficients for physicochemical properties in the first-stage detect/nondetect model (blue) and the second-stage air concentration model (orange). Length of thin bars corresponds to the 95% credible interval, length of thick bars corresponds to the interquartile range, and white dots denote the mean value. Two-way interaction terms are denoted with an “x” between terms and use the following abbreviations: log p = log octanol–water partition coefficient, bp = boiling point, log hl = log Henry’s law constant, rt = HPLC retention time, log oh = log OH rate constant, log koc = log soil adsorption coefficient.

Regression coefficients from the two stages of our model provided information on how the physicochemical properties of a substance were correlated with its odds of detection and air concentration when present, based on past sampling of US workplaces. Across all workplaces and physicochemical properties, substances were more likely to be detected if they were predicted to have a low boiling point and high log octanol–water partition coefficient, Henry’s law constant, and high-performance liquid chromatography (HPLC) retention time (significant main effects; Figure 3, blue). Substances were detected in higher concentrations when they were predicted to have a low boiling point and HPLC retention time and high Henry’s law constant (Figure 3, orange). The significant effects of boiling point are likely driven by differences in vapor pressure, and thus chemical volatility. In the case of predicted HPLC retention time, there are likely physicochemical or structural factors driving this property that also correlate with chemical use patterns and/or volatility. For example, chemicals with high volatility tend to have longer retention times than those of similar molecular weight but low volatility. In addition to these overall trends, there were several significant two-way interactions between physicochemical properties. For example, substances with a high predicted HPLC retention time were much more likely to be detected, and at higher air concentrations, when they also had high predicted soil absorption coefficients (rt × log koc, Figure 3).

### Relationship between Industry Classification and Workplace Air Monitoring

**Figure 4:**
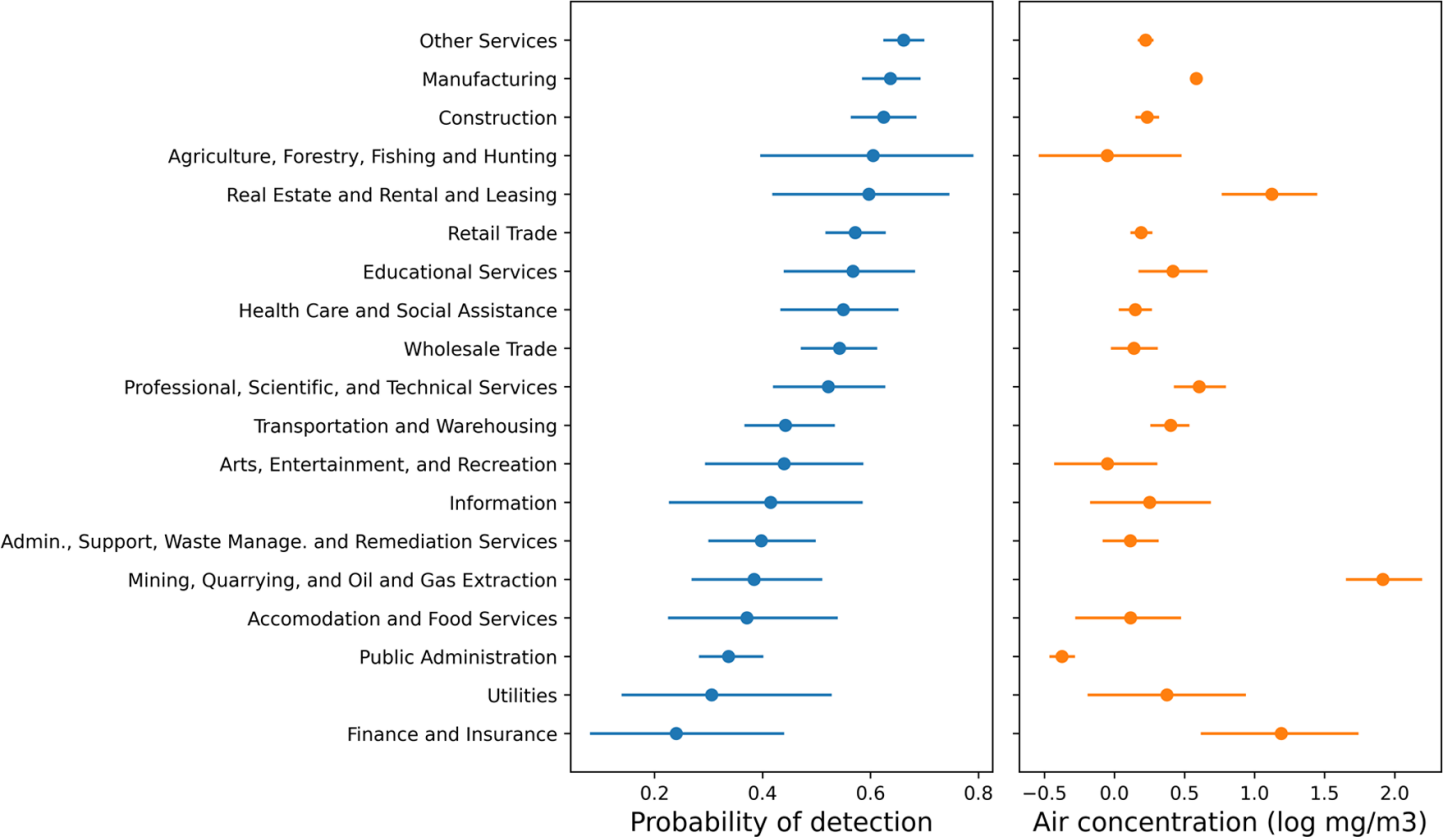
Model predictions for the probability of detecting chemicals in workplace air samples and the concentrations (log_10_ mg m^–3^) observed when a chemical was detected for each NAICS sector of industry. Predictions are for the full OSHA dataset (training + test sets). Dots denote median predictions, and bars denote 95% prediction intervals.

Detection frequency and air concentration of chemicals in workplace air samples varied strongly by industry classification. The three NAICS sectors where chemicals were most likely to be detected in OSHA workplace air samples were “Other Services”, “Manufacturing”, and “Construction”, where the median predicted detection frequency was above 62% (Figure 4). “Other Services” contained the subsectors: “Personal and Laundry Services”, and “Repair and Maintenance”. The three sectors with the lowest detection probability were “Finance and Insurance”, “Utilities”, and “Public Administration”, which had median predicted detection frequencies below 35%. The distributions of air concentrations for detected chemicals did not follow the same trend as detection frequency. For example, “Mining, Quarrying, and Oil and Gas Extraction” had the highest median predicted air concentration at 91 mg m^–3^, despite having a relatively low detection frequency of 38% (Figure 4).

The three subsectors where chemicals were most likely to be detected in workplace air samples were: “Leather and Allied Product Manufacturing”, “Furniture and Related Product Manufacturing”, and “Support Activities for Agriculture and Forestry”, which had detection frequencies just above 80% (Figure S1). These three subsectors also had the highest detection frequencies in the empirical data, at 81, 81, and 100%, respectively. The three subsectors where chemicals were predicted to be observed in the highest concentrations when detected in workplace air were: “Support Activities for Mining”, “Leather and Allied Product Manufacturing”, and “Real Estate”, which had median predicted air concentrations of 82, 42, and 34 mg m^–3^, respectively. For comparison, mean air concentrations for these subsectors in the empirical data were 82, 67, and 56 mg m^–3^, respectively.

### Estimating Occupational Exposure for New Substances

To demonstrate how this modeling framework could be applied to prioritize new substances for further study, we grouped our predictions for the test set by substance and ranked this list by detection frequency and median air concentration across all NAICS sectors and subsectors (Figure S2 and Table S1). Of the test set substances that were sampled by OSHA at least five times, 1,1,2-trichloro-1,2,2-trifluoroethane (a chlorofluorocarbon), 3-heptanone (a fragrance and solvent), and 2-ethylhexyl acrylate (an adhesive and binding agent) had the highest predicted median detection probability. The substances with the highest predicted median air concentration were: 1,1,2-trichloro-1,2,2-trifluoroethane (a chlorofluorocarbon), isobutane (a common solvent and propellant), and sevoflurane (an inhalational anesthetic). Their rankings based on empirical median air concentrations when detected were 1/27, 6/27, and 13/27. The substance with the highest predicted and actual median air concentrations, 1,1,2-trichloro-1,2,2-trifluoroethane, was detected by OSHA primarily in the “Manufacturing” sector (*n* = 88) but also in “Public Administration”, “Other Services”, “Professional, Scientific, and Technical Services”, “Finance and Insurance” and “Information” (all *n* < 6). Because 1,1,2-trichloro-1,2,2-trifluoroethane had significantly higher empirical and predicted air concentrations than other substances in the test set and was highly sampled by OSHA, it appears as a separate group of points in the top right of the plot of actual vs predicted concentrations (Figure 2b). Isobutane was detected in the sectors “Manufacturing” (*n* = 3), “Other Services” (*n* = 1), and “Professional, Scientific, and Technical Services” (*n* = 1). Sevoflurane was detected in the sectors “Health Care and Social Assistance” (*n* = 2) and “Professional, Scientific, and Technical Services” (*n* = 1). We also generated model predictions for the entire OSHA dataset (training + test sets) for further reference (Table S2).

We also applied our predictive model to 5583 new substance-by-NAICS sector/subsector pairs reported in the US EPA CDR dataset. For each pair, we used our model trained on OSHA data to predict the detection probability and distribution of air concentrations when detected (Figure S3). The 15 substance-by-workplace pairs with the highest predicted median air concentration were dominated by the “Manufacturing” sector and “Chemical Manufacturing” subsector (Table S3). Of the 11 substances represented in the top 15 pairs, there were 6 per- and polyfluoroalkyl substances (PFAS), as defined by their inclusion in the US EPA CompTox Dashboard “PFAS structures in DSSTox” list.^41^ The full set of CDR predictions is available in Table S4.

## Discussion

Our model results illustrate how occupational exposure varies by chemical properties and industry type, and how these trends can be harnessed to estimate workplace air exposure for novel substances. We have explicitly used the hierarchical nature of the workplace coding system to account for correlation between similar industry types and inform the estimates of detection frequency and air concentrations presented here. The two-level model structure further facilitates these estimates by accounting for the large number of nondetects among the observations.

### Model Performance and Use of Predictions

When using a held-out set of workplace air samples as a surrogate for novel substances, we were able to predict whether a substance would be detected in samples at a given workplace type with a high degree of accuracy (∼76%), despite the likelihood that air concentrations vary both temporally and spatially, even within a single sampling location. Additionally, OSHA inspections may or may not be triggered by known or suspected unsafe air releases and samples could span pre-and post-remediation of releases,^42^ and our model performs reasonably well despite not being able to account for these sources of variation. Estimating the magnitude of air concentrations for detects was a more challenging task given that observations varied across ∼11 orders of magnitude. Possible sources of prediction error included unaccounted variation in physical conditions and chemical use patterns across workplaces within the same industry type, temporal trends in chemical use patterns, sampling methods, and analytical techniques, and error in physicochemical property predictions. Despite these challenges, for 57% of the held-out test set samples, our hurdle model was able to correctly predict detection or nondetection and predict the detected air concentration within 1 order of magnitude. For comparison, a null model based only on the mean detection probability and air concentration of the training set achieved this level of accuracy on only 35.2% of test set samples. On the test set, the 95% prediction intervals for each sample spanned an average of 3.7 orders of magnitude. Our RMSE on untrained data of about 1 order of magnitude is roughly comparable to the scale of errors for a meta-model of chemical exposure from near-field, dietary, and fair-field pathways^14^ and may be sufficient for chemical screening and prioritization efforts.

The outputs of our model are predicted distributions of workplace air concentrations, which can be used as inputs for inhalation models that estimate worker exposure in terms of dose. For example, various inhalation models in the EPA ChemSTEER tool take air concentration as an input, along with parameters related to worker behavior and physiology (e.g., body weight, exposure frequency, and duration) and produce estimates of dose in mg kg^–1^ BW day^–1^ over various time frames.^23,24^ For substances where data on the typical distribution of air concentrations that might be present at a job site is lacking, models may be run assuming the substance is present in the air at the permissible exposure limit (PEL), if one is available.^25,43^ Our modeling framework could be used to replace these PEL assumptions by generating estimates of air concentration, in the form of a probability distribution, for such substances with little to no empirical data. Thus, these probability distributions, combined with estimates of worker behavior and physiology, can serve as inputs to worker exposure models that generate dose ranges.

We generated predictions for the OSHA test set and the US EPA CDR data to demonstrate how this statistical framework could be applied to estimate the workplace air exposure potential of new substances or substance-by-workplace pairs. We chose not to extrapolate beyond the original workplace types present in the OSHA dataset and included in our hierarchical model (Table S5), although it should be possible to make predictions for such data by setting the subsector random intercept or sector hyperprior to zero, effectively assuming that the effect of a novel sector or subsector is equal to the average of those already observed. Although the model was not trained on any of the OSHA test set substances, nor most of the CDR substances, it benefits from data on substances that have similar physicochemical properties and were observed in similar workplaces in the training data. To prioritize these substances based on the likelihood of risk, these estimated air concentrations must be compared to each substance’s hazard, i.e., what level of exposure is likely to produce a negative health outcome.^3^ For example, 1,1,2-trichloro-1,2,2-trifluoroethane was the substance in the test set that was most frequently detected and in the highest air concentration, when sampled by OSHA, but its median predicted air concentration of 3660 mg m^–3^ was well below the OSHA permissible exposure limit of 7600 mg m^–3^^43^ and the level considered immediately dangerous to life or health of 15 200 mg m^–3^.^44^ However, for many substances regulated by TSCA, information on what air concentration results in health hazards is not available. In these cases, new approach methodologies such as high-throughput bioactivity screening and high-throughput toxicokinetics models can be used to estimate the hazard in terms of dose. To compare our results to these hazard predictions, our air concentration predictions could be converted to worker dose (e.g., mg kg^–1^ day^–1^) using a worker exposure model such as those in the EPA ChemSTEER tools. Substances could then be sorted by the difference between estimated exposure and estimated hazard, both in terms of dose, to identify those with a higher likelihood of risk for further scrutiny.^3^ Focused investigations into the identified substances should consider critical context such as the amount of the substance manufactured/used/stored in typical workplaces and the activity patterns and PPE use of workers.

One challenge when applying this framework to predict occupational exposure for a novel substance with no sampling data is that we may lack information on what types of industry (NAICS sector and subsector) may utilize this substance at present or in the future. In these cases, chemical functional use models, machine-learning-based models that classify substances into their likely functional roles in products and processes, represent a potential tool for mapping the chemical structure to a number of candidate industries.^5,45^ Air exposure could then be estimated for each candidate industry type using our data-driven statistical framework that leverages historical air monitoring data for each specific industry sector and subsector.

While the model presented here can be used to make predictions for chemicals not present in the OSHA database, a second application may be in using the model’s predictions for chemicals within the database as reference values by chemical and industry, for evaluation of other model estimates. That is, there are several challenges to using all of the observations of a given chemical for a given industry within the OSHA database for statistical evaluation, such as the small number of observations in certain categories and the high rate of nondetects. The estimates of our model might be thought of as a summary of the OSHA observations, allowing for a more direct analysis of differences between predicted and observed air concentrations. The results of this model might be used to systematically evaluate, calibrate, and develop consensus occupational exposure models as in the EPA’s Systematic Empirical Evaluation of Models (SEEM).^3^

### Limitations

Although the OSHA Chemical Exposure Health Data is, to our knowledge, the most comprehensive publicly available database of workplace chemical monitoring, there are inherent limitations to this dataset that must be considered. While some workplace inspections are carried out randomly within high-hazard industries, many are triggered by complaints of possible violations or worker health incidents.^42^ As a result, these data may be biased toward higher-than-average chemical exposure scenarios, particularly in traditionally low-hazard industries where random inspections for compliance are less common. This possible source of bias, plus the fact that we used the maximum concentration from each sampling event, may produce estimates that are more representative of “worst-case scenarios,” but a consideration of such high-risk incidents is useful in the context of chemical risk screening and prioritization.^46^ Additionally, physical conditions at workplaces vary considerably, for example, indoor vs outdoor areas, room size, and ventilation rate, and the OSHA dataset lacks such information. However, the sector and subsector random intercepts in the model can account for mean differences between workplace types, and thus our air concentration estimates should be considered to reflect the mean physical conditions of each sector and subsector as sampled by OSHA.

Because the OSHA dataset spans more than three decades of compliance activities, it is likely to include a number of temporal trends in industrial activities that our model could not account for. From 1984 to the present, there have been changes in the types of industrial processes carried out, the procedures and protections involved in these processes, and the types of substances being used and produced. These patterns are difficult to account for in a broad screening model, as they have occurred simultaneously and likely affect each substance differently. For example, 1,1,2-trichloro-1,2,2-trifluoroethane (also known as CFC-113), was predicted to be most frequently detected and with the highest air concentration out of the test set chemicals, but it has been completely phased out of production and importing in the United States since 1996, in accordance with the Montreal Protocol. As a result, 106 of the 108 OSHA samples for this substance occurred before 1996. While estimates of present-day exposure risk need to consider such temporal trends, inclusion of these historical data in the model helps to inform the relationship between the physicochemical properties of substance and workplace air concentrations. Future model development could also consider a weighting system where more recent observations are given more influence in the model fitting process.

A further challenge when analyzing this dataset is the lack of information on the detection limit for each air sample. As such, it is not possible to determine whether a chemical was truly not present in a sample or if it was simply below the assay’s limit of detection, and zero values cannot be substituted with placeholder values based off the detection limit (e.g., half the limit of detection). This also precludes the use models that account for limit of detection, such as censored regression models. Further, with the dataset spanning more than 30 years of sampling, it is reasonable to assume that the detection limit varied over time even for individual chemicals, as analytical chemistry methods and instrumentation have advanced. This uncertainty and temporal variation in the limit of detection may contribute to our model’s systematic misclassification of many low-concentration measurements as nondetects rather than detects (Figure 2). Despite the challenges inherent to a broad modeling approach trained on a complex dataset, even a rough prediction of workplace air exposure potential, combined with an associated estimate of uncertainty, can be of use when prioritizing a large number of substances for further scrutiny.^46^

### Implications for Risk Assessment

There are little to no data available on occupational use patterns, production amounts, or air releases for many chemicals, which limits our ability to perform risk assessment on the tens of thousands of substances used in commerce. We present a data-driven approach that leverages over three decades of workplace samples to model air concentrations as a function of industry type and the physicochemical properties of a substance. This model dramatically outperforms a null model when predicting whether a substance will be detected in an air sample, and if so at what concentration. Predicted air concentrations from this model can be used as inputs to exposure models to estimate worker doses and could be combined with high-throughput exposure estimates for other pathways such as ambient air, consumer product and dietary sources to build a more complete picture of individual exposure. This type of exposure information should be paired with an understanding of hazard from high-throughput bioactivity assays and/or pharmacokinetics models to identify and prioritize substances that may pose high risk, i.e., where exposure is predicted to be close to the levels that may be hazardous to human health. These new approach methodologies can be used in tandem with traditional risk assessment tools to meet the challenge of identifying high-risk chemicals and protecting human health in an ever-growing chemical space.

## Data Availability

Input data and code to recreate these analyses are provided at: https://github.com/USEPA/ht_occupational or https://doi.org/10.5281/zenodo.7737239.

## Supporting Information Available

Additional figures and information about US EPA CDR data processing (PDF)
Additional tables describing model inputs and predictions (XLSX)
