# Supplementary material for: A Data-Driven Approach to Estimating Occupational Inhalation Exposure Using Workplace Compliance Data
Source: Environ Sci Technol. 2023 Mar 30;57(14):5947–56. doi: 10.1021/acs.est.2c08234 (PMC10100548; doi:10.1021/acs.est.2c08234)
Supplement: Supplementary file 1 — es2c08234_si_001.pdf [file es2c08234_si_001.pdf]

Supporting Information for:

A data-driven approach to estimating occupational inhalation exposure using workplace compliance data

Jeffrey M. Minucci[1], S. Thomas Purucker[2], Kristin K. Isaacs[2], John F. Wambaugh[2], and Katherine A. Phillips[2]*

[1] *US Environmental Protection Agency, Office of Research and Development, Center for Public Health and Environmental Assessment, Durham, NC, 27709, USA*

[2] *US Environmental Protection Agency, Office of Research and Development, Center for Computational Toxicology and Exposure, Durham, NC, 27709, USA*

** Corresponding author. Email: minucci.jeffrey@epa.gov Address: 109 TW Alexander Dr, Durham, NC 27709, USA*

Pages: 9

Figures: 3

Table of Contents

Section S1 3
Section S2 6
Figure S1 6
Figure S2: 7
Figure S3: 8
References 8

Section S1

To demonstrate how our model could be used to screen new substance and workplace combinations without OSHA monitoring data, we leveraged data from the US EPA’s Toxic Substances Control Act (TSCA) Chemical Data Reporting (CDR) database. The data under the US EPA’s CDR rule is collected in four-year cycles. The most recent cycle which was publicly available at the time of our analysis was the 2016 cycle. The comma separated values (CSV) file of the 2016 CDR was obtained from the US EPA's CDR download page.[1] We used the industrial use file from the 2016 CDR cycle as it was considered most relevant to the field of occupational exposure and was the only reporting file that contained information on industrial sectors.

To make predictions, our model required the NAICS sector and subsector codes (*i.e.*, the first 3 NAICS code digits) and the chemical structure in the form of a SMILES string, which is passed to the OPERA QSAR models to obtain the necessary physicochemical properties. While chemical and industrial use information is required to be reported to the US EPA, this information can be redacted from the public version of the data if it is considered confidential business information (CBI), which leads to many records in the public database being unusable. In other cases, due to the manufacturing pipeline, industry type may not be known or reasonably ascertainable and may be declared as such. Further, when reporting data, reporters must choose one of 48 US EPA industrial sector (IS) codes for each record to convey the industry that uses the substance. The US EPA does provide a crosswalk of IS codes to NAICS codes when discussing the reasons for switching from requiring manufacturers to report NAICS codes to requiring IS codes.[2] However, the 48th industrial sector code (IS48) is essentially a code that allows a CDR reporter to insert free text into this field. For this reason, matching the text in the IS code field is not a straightforward process.

The CDR industrial use file for the 2016 cycle contains 64,389 records of reported chemical information. Records whose chemical substance or industrial sectors was either redacted as CBI or left blank were removed. For the remaining records, the text for the industrial codes was canonicalized to facilitate matching using the IS-to-NAICS crosswalk. After this, only unique records of Chemical Abstracts Service Registry Number (CAS RN), chemical name, and industrial sectors were retained. This left a dataset of 24,757 records with 7,382 unique CAS RNs and 544 unique industrial sectors. An attempt was made to match all chemical names and CAS RNs in this dataset to DTXSIDs via the US EPA's CompTox Chemicals Dashboard's batch search functionality.[3] We dropped any records for substances that could not be matched to DTXSIDs, or did match but did not have associated QSAR-ready SMILES strings, as there would be no way to obtain the necessary physicochemical properties from the OPERA QSAR suite. After matching, there were 12,512 records with 3,702 unique CAS RNs and 215 unique industrial sectors. The 215 unique sectors were matched to the IS-to-NAICS code crosswalk, which provided 54 direct matches. The rapidfuzz Python package[4] was then used to perform sorted, tokenized matching of the remaining 161 IS codes to NAICS codes. In some cases, it was found that the free text of the IS code field mapped to multiple NAICS codes. When this occurred, the record was split into multiple records so that each record contained only a single substance by NAICS code pair.

After fuzzy text matching and expansion, the dataset contained 3,701 unique substances and 15,152 unique substances by NAICS code pairs. We then eliminated any records with NAICS sector and subsectors that were outside the domain of our OSHA dataset (Table S2) since our model could not make predictions for these observations. Additionally, 341 of the substances from the CDR data had OPERA-predicted physicochemical properties that were

outside the domain of the OSHA data our model trained on (Table S3), so we removed these substances to avoid extrapolation. Finally, we removed substance-by-NAICS sector/subsector combinations that were already included in the OSHA data analysis. This resulted in a final dataset of 2,875 new substances and 5,583 new substance-by-NAICS sector/subsector pairs. Of the 7 NAICS sectors represented in the final dataset, "Manufacturing" was by far the most common, with ~96% of reports falling into this category. Each unique pair was passed to our pre-trained two-part Bayesian hierarchical model in the form of NAICS sector and subsector and the six required physicochemical properties and their two-way interactions. For each pair, our model predicted a detection probability and an air concentration for detects.

Section S2

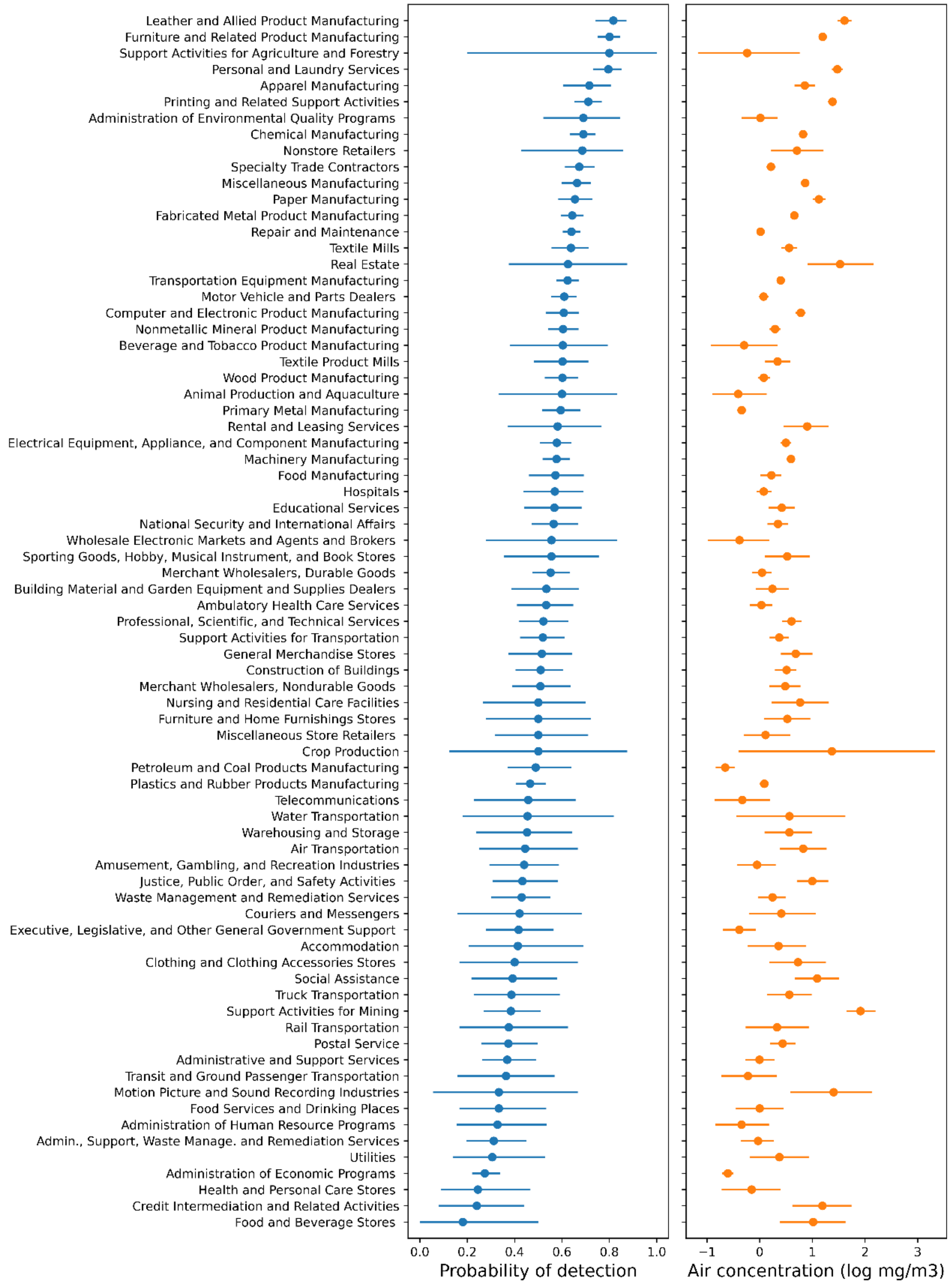

Figure S1: Model predictions for the probability of detecting chemicals in workplace air samples (left) and the concentrations observed when a chemical was detected (right) for each

NAICS subsector of industry. Dots denote median predictions and bars denote 95% prediction intervals.

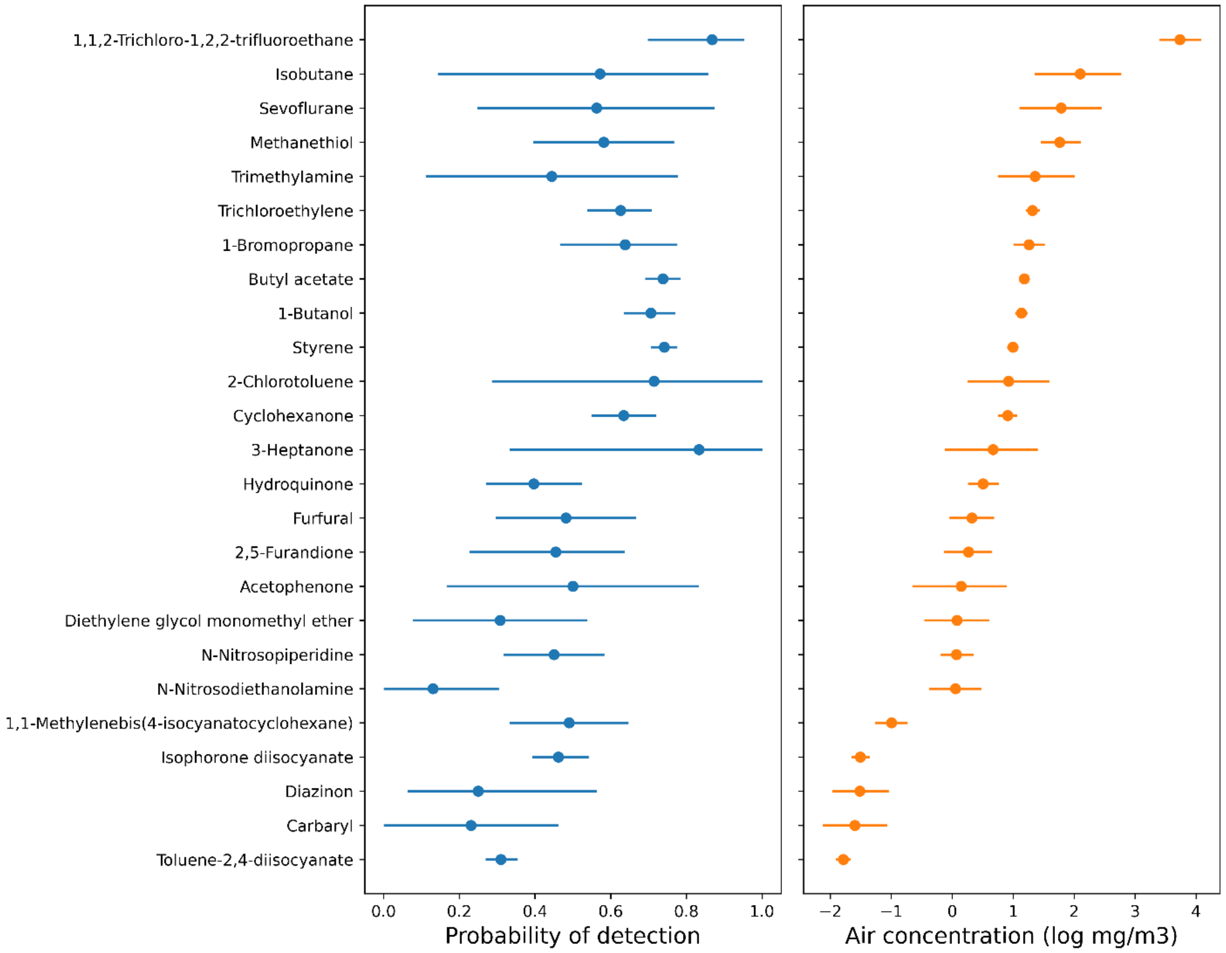

Figure S2: Model predictions of detection probability (left) and air concentration distribution (right; $\log_{10}$ mg m^{-3}) for the substances present in the test set of OSHA workplace air data (observations ≥ 5). Dots denote median predictions and bars denote 95% prediction intervals.

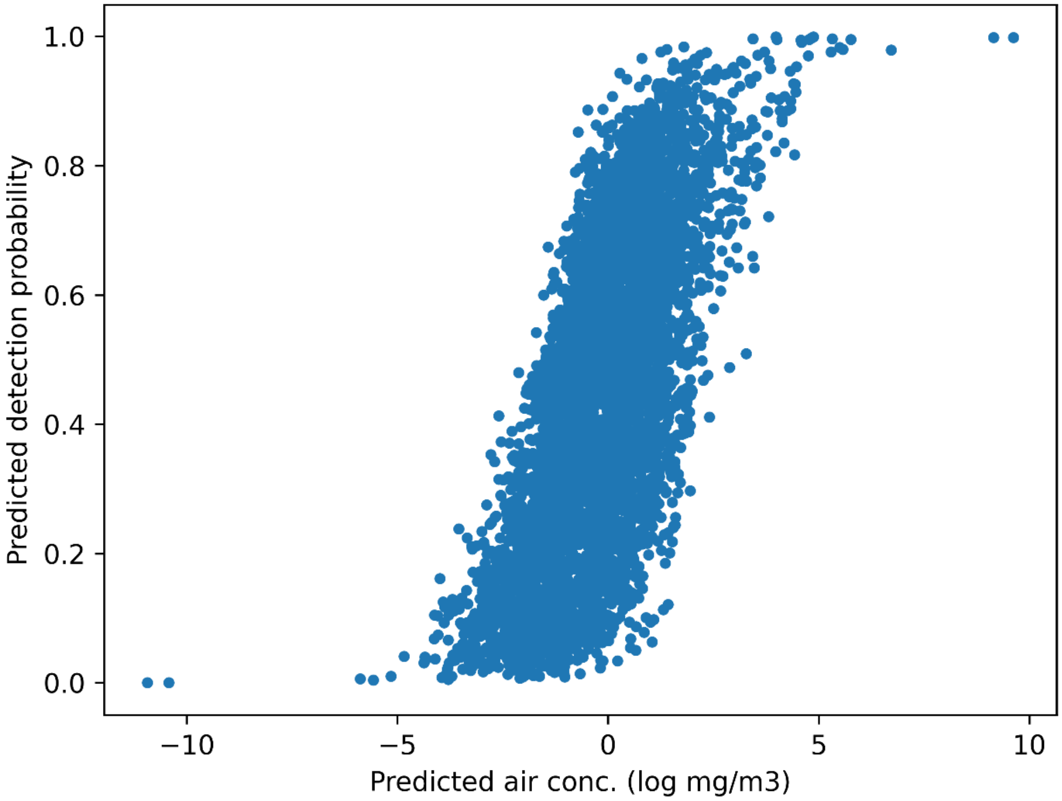

Figure S3: Plot of predicted detection probability and median air concentration ($\log_{10}$ mg m^{-3}) for 5,583 new substance-by-workplace pairs reported in the US EPA's Toxic Substances Control Act (TSCA) Chemical Data Reporting (CDR) industrial use database from the 2016 cycle.

References

1. USEPA *Chemical Data Reporting, 2016 cycle*; 2020; https://www.epa.gov/chemical-data-reporting/access-cdr-data.

2. USEPA *Replacement of 5-digit NAICS Codes with Industrial Sector Codes*; 2022; https://www.epa.gov/chemical-data-reporting/replacement-5-digit-naics-codes-industrial-sector-codes.

3. Lowe, C. N.; Williams, A. J., Enabling High-Throughput Searches for Multiple Chemical Data Using the U.S.-EPA CompTox Chemicals Dashboard. *Journal of Chemical Information and Modeling* **2021,** *61*, (2), 565-570. DOI: 10.1021/acs.jcim.0c01273

4. Bachmann, M. *maxbachmann/RapidFuzz: Release 1.8.1*, v1.8.1; Zenodo, 2021. DOI: 10.5281/zenodo.5590421
